# In Vitro and In Vivo Characterization of 40 kDa PEGylated Adrenomedullin in a DSS-Induced Colitis Model

**DOI:** 10.3390/ijms26199373

**Published:** 2025-09-25

**Authors:** Kazuo Kitamura, Emiko Akashi, Sayaka Nagata, Toshihiro Kita, Motoo Yamasaki

**Affiliations:** 1 Department of Project Research, Frontier Science Research Center, University of Miyazaki, Miyazaki 889-1692, Japan; emiko_akashi@med.miyazaki-u.ac.jp (E.A.); sayaka_nagata@med.miyazaki-u.ac.jp (S.N.); toshihiro_kita@med.miyazaki-u.ac.jp (T.K.); motoo_yamasaki@med.miyazaki-u.ac.jp (M.Y.); 2 Department of Pharmacy, University of Miyazaki Hospital, Miyazaki 889-1692, Japan; 3 Department of Food Science and Technology, Faculty of Health and Nutrition, Minami Kyushu University, Miyazaki 880-0032, Japan; 4 Himuka AM Pharma Corp., Miyazaki 880-0812, Japan

**Keywords:** adrenomedullin (AM), 40 kDa PEGylated AM (PEG-AM), receptor, cAMP, inflammatory bowel disease, colitis, dextran sodium sulfate (DSS)

## Abstract

Adrenomedullin (AM), a biologically active peptide, induces complete remission with mucosal healing in patients with refractory ulcerative colitis. We have developed 40 kDa PEGylated AM (PEG-AM), a long-acting AM derivative, as a potential therapeutic agent for inflammatory bowel disease (IBD). Both PEG-AM and native AM stimulated cyclic adenosine monophosphate (cAMP) production in HEK-293 cells stably expressing the AM1 receptor (CLR/RAMP2 complex), in a dose-dependent manner. The pEC_50_ values for PEG-AM and AM were 7.23 ± 0.05 and 8.42 ± 0.10, respectively. PEG-AM exhibited significantly greater stability in plasma and serum than native AM. We evaluated the in vivo anti-colitis effects of intravenously administered PEG-AM in a dextran sodium sulfate (DSS)-induced murine colitis model. A single intravenous dose of PEG-AM, as low as 25 nmol/kg, demonstrated therapeutic efficacy. Notably, AM receptor expression was not downregulated, despite sustained high plasma concentrations of PEG-AM. Additionally, PEG-AM exerted both therapeutic and preventive effects in a DSS colitis model. These findings suggest that PEG-AM is a promising therapeutic candidate for the treatment of patients with IBD.

## 1. Introduction

Ulcerative colitis (UC) and Crohn’s disease (CD) are the most common forms of chronic inflammatory bowel disease (IBD) [1]. The exact cause of IBD remains unclear, but it is believed to involve abnormal intestinal immunity, genetic predisposition, and environmental factors, including diet and intestinal microflora [2]. Although there are currently no drugs that can completely cure IBD, remarkable progress has been made over the past two decades in the development of therapeutic agents that can induce and maintain remission. These agents include anti-TNFα antibodies, tacrolimus, anti-IL-12/23p40 antibodies, Janus kinase inhibitors, and integrin inhibitors [3,4]. Although these drugs show efficacy, they can compromise the patient’s immune system, sometimes leading to infections and malignant lymphomas. Furthermore, biologics are immunogenic and may be targeted by anti-drug antibodies [5,6], which result in a reduced therapeutic effect, a phenomenon known as secondary failure. Therefore, there is a need for new therapeutic agents with novel mechanisms of action for treatment of IBD.

Adrenomedullin (AM) is a bioactive peptide with vasodilatory activity originally isolated from human pheochromocytomas [7]. It consists of 52 amino acid residues, has an amidated C-terminus, and forms a ring structure via intramolecular disulfide bonds. These structural features are essential for AM to bind to its receptors and perform its biological functions. The N-terminal region of AM is not involved in its biological activity [8,9].

AM and calcitonin gene-related peptide (CGRP) share partial amino acid sequence homology and belong to the AM/CGRP superfamily [10]. AM and CGRP receptors are heterodimeric complexes composed of a calcitonin receptor-like receptor (CLR) and one of three receptor activity-modifying proteins (RAMP1, RAMP2, or RAMP3) [11]. CGRP binds with high affinity to the CLR/RAMP1 complex, whereas AM binds with low affinity to this complex. Instead, AM binds to CLR/RAMP2 (AM1 receptor) and CLR/RAMP3 (AM2 receptor) complexes, with the CLR/RAMP2 complex considered the primary receptor mediating AM function [10,12,13]. AM-specific binding sites are widely distributed throughout the body, with particularly high densities in the lungs, kidneys, cardiovascular tissues, and gastrointestinal tract [14,15].

AM has shown therapeutic effects in animal models of various diseases, including cardiovascular diseases, renal disorders, and inflammatory bowel disease (IBD) [16,17]. In particular, continuous intravenous administration of AM has demonstrated both efficacy and safety in patients with ulcerative colitis and Crohn’s disease [18,19]. The mechanism by which AM ameliorates intestinal inflammation is thought to involve multiple actions, including anti-inflammatory effects, promotion of intestinal epithelial repair, and enhancement of angiogenesis and lymphangiogenesis [20,21]. Notably, the direct regenerative effect of AM on intestinal epithelium is a unique feature absent in existing IBD drugs [22,23,24]. We believe that this action will lead to a high probability of complete remission in UC patients treated with AM [18,20].

AM possesses several desirable pharmacological properties for use as a therapeutic agent for IBD, including high receptor specificity and potent receptor activation [20,25]. However, its rapid and strong hypotensive effect can cause adverse reactions, including increased heart rate and compensatory sympathetic activation [26,27]. Therefore, AM administration currently requires hospitalization and continuous infusion with careful dose adjustments. Other limitations include poor pharmacokinetic profile due to rapid proteolytic degradation and clearance from the circulation.

To improve the convenience and pharmacokinetic stability of AM, we investigated various polyethylene glycol (PEG) AM derivatives with PEG attached to the N-terminus. Previous studies demonstrated that 5 kDa, 20 kDa, and 60 kDa PEG-AM derivatives prolonged the half-life of AM but showed progressively reduced receptor affinity [28,29,30]. Of the PEG-AM derivatives examined, the 40 kDa form provided the most favorable balance between potency and pharmacokinetic stability. Furthermore, 40 kDa PEG represents the most widely established high–molecular weight PEG in marketed pharmaceuticals [31,32], offering both regulatory precedent and clinical experience that support its selection as a development candidate.

In the present study, we describe in vitro biological activity and in vivo pharmacological effects of PEG-AM in a DSS-induced colitis model. These data are essential for clinical development of PEG-AM.

## 2. Results

### 2.1. Synthesis and Characterization of PEG-AM

**Figure 1:**
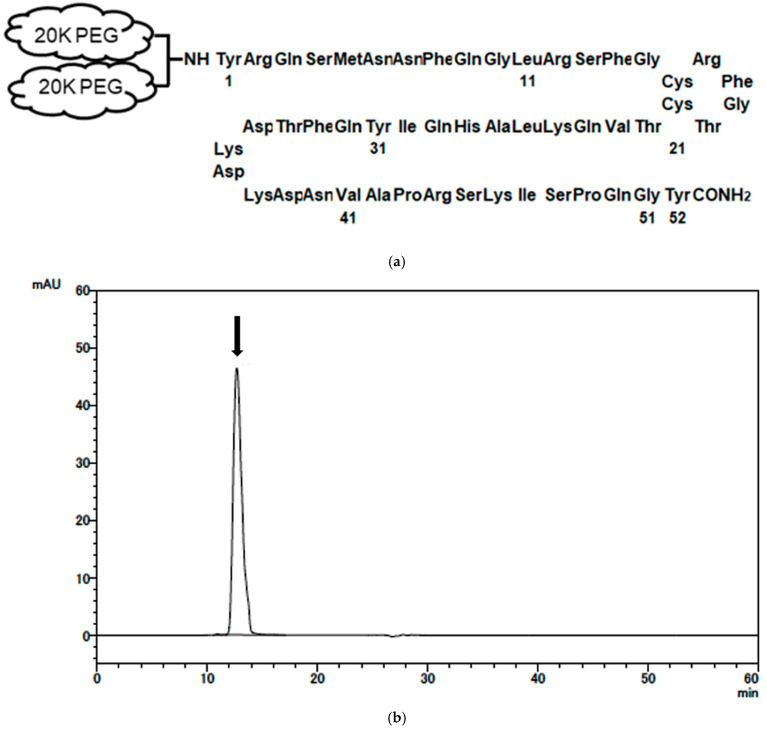
Structure and characterization of mono-PEGylated adrenomedullin (PEG-AM). (**a**) General structure of PEG-AM. The N-terminal tyrosine of human AM is conjugated to a PEG aldehyde reagent (NOF Co., SUNBRIGHT LY-400AL3). The PEG moiety consists of a 40 kDa, two-armed, lysine-branched structure (2 × 20 kDa). (**b**) Gel filtration chromatogram of PEG-AM. Column: Superdex 200 Increase 10/300 GL (10 mm × 300 mm, GE Healthcare). Eluent: 20 mM trisodium citrate buffer (pH 7.2) containing 1.0 M NaCl. Flow rate: 0.75 mL/min. Sample: 0.05 mg/0.01 mL of PEG-AM. Absorbance at 280 nm was monitored. The arrow indicates the elution peak of mono-PEGylated AM at 12.8 min. Total analysis time 60 min.

AM was modified by conjugation with a 40 kDa PEGylating reagent (SUNBRIGHT^®^ LY-400AL3). The structure of PEG-AM is shown in Figure 1a. In the 40 KDa PEG, the two 20 kDa PEG branches are linked by a lysine molecule, also called PEG-linker lysine. AM contains four lysine residues, at positions 25, 36, 38, and 46, all of which are potential PEGylation sites. However, PEG binds exclusively to the N-terminal tyrosine residue of the AM peptide and not to the ε-amino groups of lysine residues (Figure 1a). CNBr digestion of PEG-AM followed by ion-exchange chromatography confirmed exclusive PEGylation at the N-terminal tyrosine residue, as the AM (6–52) fragment was recovered intact.

Figure 1b shows the results of gel filtration chromatography. The PEG-AM appeared as a single peak and authentic mono-PEGylated adrenomedullin (PEG-AM) was eluted within 12.8 min. DiPEGylated adrenomedullin (DiPEG-AM) elutes at 11.4 min, but no such peak was observed.

### 2.2. In Vitro Biological Activity of PEG-AM

**Figure 2:**
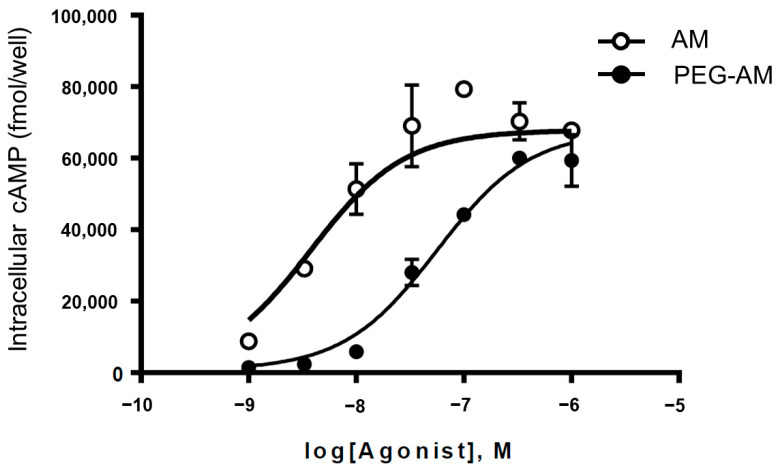
Intracellular cAMP accumulation induced by PEG-AM or human adrenomedullin (AM) in HEK-293 cells stably expressing the AM type I receptor (AM1 receptor). Data are presented as mean ± SEM (*n* = 4 per group).

The biological activities of AM and PEG-AM were evaluated in HEK-293 cells stably expressing calcitonin receptor-like receptor (CLR) and receptor activity-modifying protein 2 (RAMP2), which form the AM1 receptor. AM and PEG-AM stimulated intracellular cyclic adenosine monophosphate (cAMP) accumulation (Figure 2). AM increased cAMP levels in a dose-dependent manner, with a pEC_50_ value of 8.42 ± 0.10. PEG-AM also increased cAMP levels, with a slightly lower potency (pEC_50_ = 7.23 ± 0.05).

### 2.3. Stability of PEG-AM and AM

**Figure 3:**
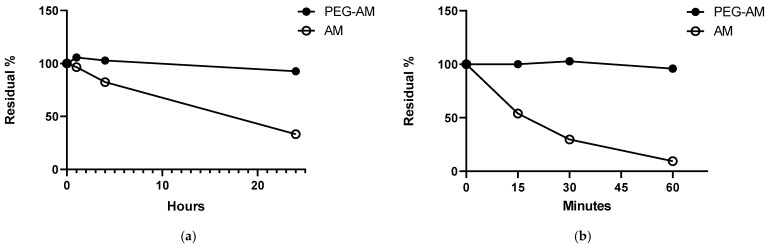
Stability of PEG-AM and AM in plasma and serum. (**a**) Degradation of PEG-AM and AM in human plasma. (**b**) Degradation of PEG-AM and AM in human serum. Data are presented as mean ± SEM (*n* = 3 per group). Error bars are shorter than the symbol size.

We assessed the stabilities of PEG-AM and native AM in human plasma (Figure 3a) and serum (Figure 3b). In plasma, AM gradually degraded, leaving 33.3% after 24 h. In contrast, PEG-AM remained highly stable, with 92.7% remaining intact after 24 h. In serum, AM degraded more rapidly, with only 9.45% remaining after 60 min, whereas PEG-AM remained stable, with 95.9% of the compound still present at 60 min.

### 2.4. Dose-Dependent Effects of Intravenous PEG-AM

**Figure 4:**
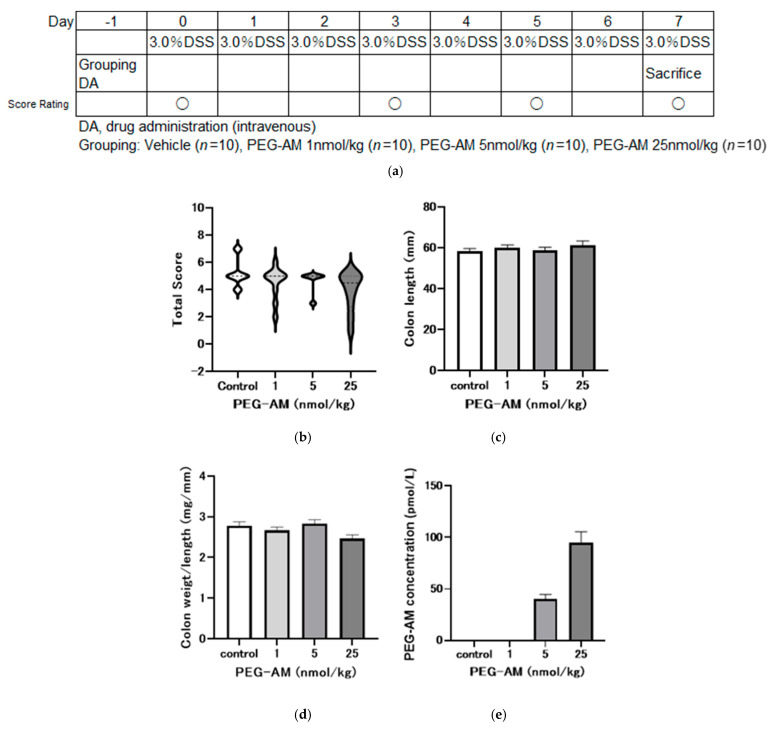
Dose-dependent effects of intravenous PEG-AM administration in DSS-induced colitis (Study No. P170454). (**a**) Overview of the experimental design. Groups: vehicle (*n* = 10), PEG-AM 1 nmol/kg (*n* = 10), 5 nmol/kg (*n* = 10), 25 nmol/kg (*n* = 10). (**b**) Total inflammation scores on day 8. (**c**) Colon length. (**d**) Colon weight-to-length ratio. (**e**) PEG-AM plasma concentrations on day 8.

We examined the effects of intravenous administration of PEG-AM on DSS-induced colitis, as per the experimental design in Figure 4a. PEG-AM was administered one day before DSS exposure to evaluate its prophylactic effect against colitis onset and progression.

Figure 4b–d present the pharmacological effects of intravenous bolus injection of PEG-AM. On day 8, no statistically significant differences in total inflammation scores were detected among the groups (Figure 4b). Colon lengths were comparable across all groups, with no significant differences (Figure 4c). Colon weight per unit length also showed no significant differences among groups, including the 25 nmol/kg group (Figure 4d).

PEG-AM plasma concentrations on day 8 were 40.53 ± 4.34 pmol/L for the 5 nmol/kg group and 94.7 ± 10.91 pmol/L for the 25 nmol/kg group (Figure 4e). PEG-AM was undetectable in the control and the 1 nmol/kg groups.

### 2.5. Effect of 25 nmol/kg PEG-AM in DSS-Induced Colitis

Based on the dose-dependent study, 25 nmol/kg was selected for subsequent experiments as it showed the most favorable numerical trends among the tested doses, despite the absence of statistical significance. The detailed rationale for this choice is provided in the Discussion. Two groups (control and treatment with 25 nmol/kg PEG-AM) were compared, each consisting of 20 animals.

**Figure 5:**
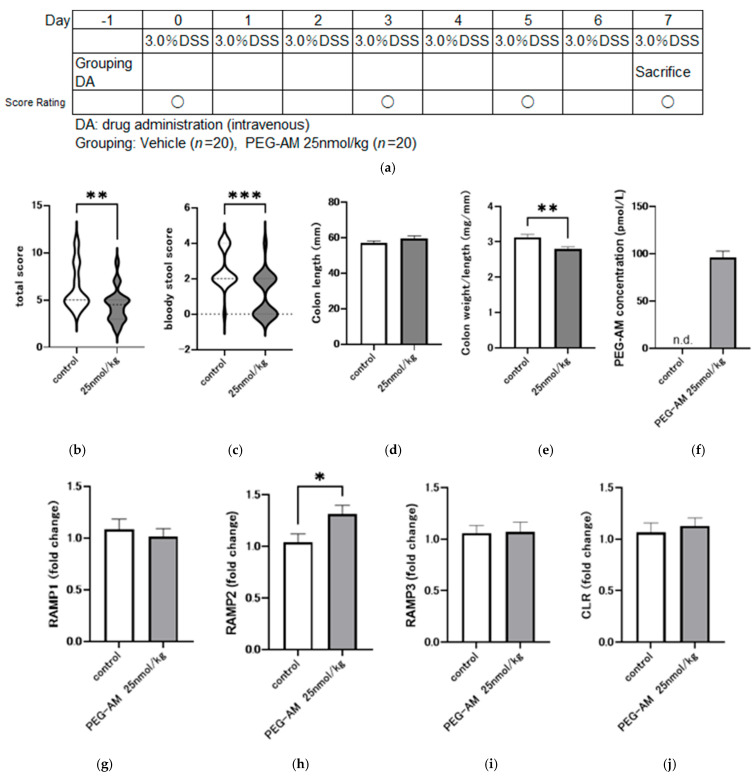
Effect of 25 nmol/kg PEG-AM in DSS-induced colitis (Study No. P180567). (**a**) Overview of the experimental design. Groups: vehicle (*n* = 20), PEG-AM 25 nmol/kg (*n* = 20). (**b**) Total inflammation scores on day 8. ** *p* < 0.01, Mann–Whitney U test. (**c**) Bloody stool scores on day 8, *** *p* < 0.001, Mann–Whitney U test. (**d**) Colon length. (**e**) Colon weight-to-length ratio, ** *p* < 0.01, unpaired *t*-test. (**f**) PEG-AM plasma concentrations on day 8. (**g**–**j**) Fold changes in gene expression of RAMP1 (**g**), RAMP2 (**h**), RAMP3 (**i**), and CLR (**j**). * *p* < 0.05, unpaired *t*-test.

Total inflammation score on day 8 was significantly lower in the PEG-AM group than in the control group (*p* = 0.0062, Mann–Whitney U test; Figure 5b). Bloody stool score was significantly lower in the PEG-AM group than in the control group (*p* = 0.0008, Mann–Whitney U test; Figure 5c). Colon length was not significantly different between groups (*p* = 0.1429, unpaired *t*-test; Figure 5d). Colon weight per unit length was significantly lower in the PEG-AM group compared to the control group (*p* = 0.0024, unpaired *t*-test; Figure 5e).

PEG-AM plasma concentration was 96.3 ± 6.72 pmol/L in the PEG-AM group and undetectable in the control group (Figure 5f). Gene expression analysis showed no downregulation of any receptor components (RAMP1, RAMP2, RAMP3, and CLR; Figure 5g–j). Notably, RAMP2 expression was significantly higher (1.3-times) in the PEG-AM-treated group compared to the control group. 

### 2.6. Therapeutic Effect of PEG-AM After Onset of DSS Colitis

Previous studies have demonstrated the preventive effects of PEG-AM administered before DSS. In the present study, we evaluated its therapeutic effects on the onset of DSS-induced colitis.

**Figure 6:**
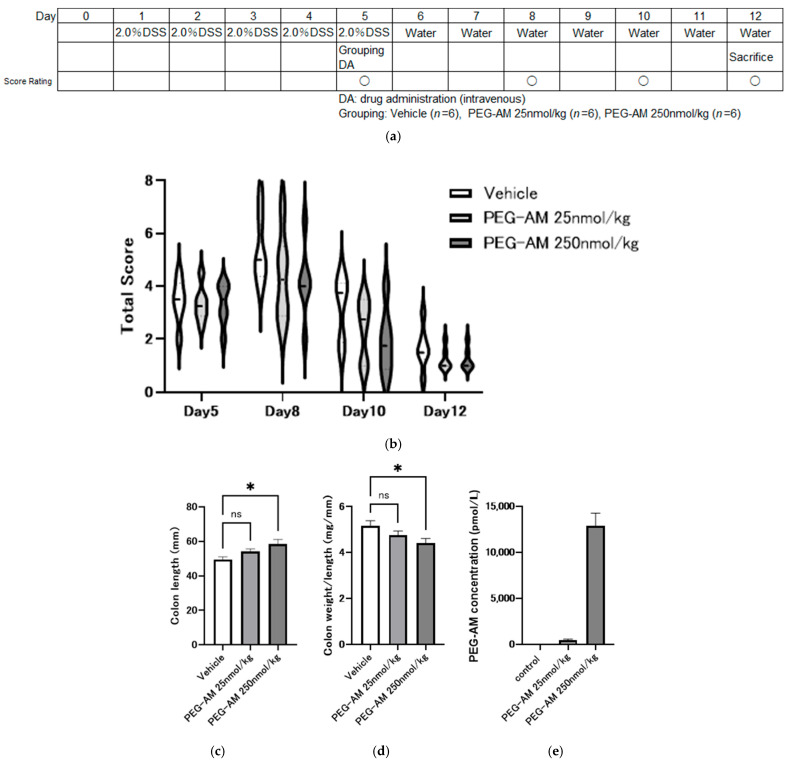

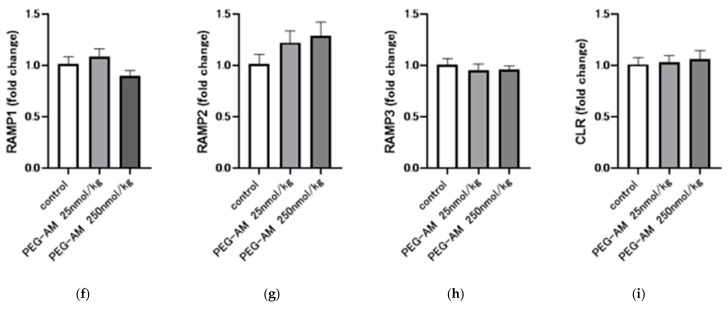
Therapeutic effects of PEG-AM administered after disease onset in DSS-induced colitis (Study No. P200487). (**a**) Overview of the experimental design. Groups: vehicle (*n* = 6), PEG-AM 25 nmol/kg (*n* = 6), 250 nmol/kg (*n* = 6). (**b**) Time course of total inflammation scores after PEG-AM administration. (**c**) Colon length, * *p* < 0.05, one-way ANOVA followed by Dunnett’s multiple-comparisons test. ns, not significant. (**d**) Colon weight-to-length ratio, * *p* < 0.05, one-way ANOVA followed by Dunnett’s multiple-comparisons test. ns, not significant. (**e**) PEG-AM plasma concentrations on day 12. (**f**–**i**) Fold changes in gene expression of RAMP1 (**f**), RAMP2 (**g**), RAMP3 (**h**), and CLR (**i**).

Figure 6a shows the experimental design. DSS was administered for five days to induce colitis, followed by the administration of PEG-AM or saline on day 5. DSS was replaced with water on day 6 to evaluate recovery. PEG-AM administration did not significantly alter total inflammation scores over time compared with the control, although numerical trends suggested lower values in treated groups (Figure 6b). The colon was significantly longer in the PEG-AM-treated group than in the control group (*p* = 0.014, one-way ANOVA with Dunnett’s test; Figure 6c). Colon weight per unit length was significantly lower in the 250 nmol/kg PEG-AM group compared to the control group (*p* = 0.039, Dunnett’s test; Figure 6d).

Plasma PEG-AM concentrations on day 12 were 473 ± 113 pmol/L in the 25 nmol/kg group and 12,911 ± 1363 pmol/L in the 250 nmol/kg group. No PEG-AM was detected in the control group (Figure 6e). No downregulation of receptor component expression was observed despite high PEG-AM plasma levels. RAMP2 expression increased in a dose-dependent manner; however, the increase was not statistically significant (Figure 6f–i).

## 3. Discussion

We described the in vitro biological activity and in vivo pharmacological effects of 40 kDa PEGylated adrenomedullin (PEG-AM) in a DSS-induced colitis model. We are currently developing PEG-AM as a therapeutic agent for IBD, and the data from this study provide a foundation for clinical development.

We assessed the in vitro activity of PEG-AM by measuring cAMP accumulation in HEK-293 cells stably expressing CLR and RAMP2, which together form the AM1 receptor —a receptor with high affinity and specificity for AM [10,33]. Both native AM and PEG-AM increased cAMP levels in a dose-dependent manner. The pEC_50_ values for native AM and PEG-AM were 8.42 ± 0.10 and 7.23 ± 0.05, respectively, indicating that PEG-AM has an approximately 15.5-fold lower affinity for the AM1 receptor compared to native AM. Previous studies have reported that 5 k, 20 k, and 60 k PEG-AM variants exhibit 2.57-, 6.60-, and 38.0-fold reduced affinities, respectively [28,29], suggesting an inverse correlation between PEG molecular weight and receptor affinity. At the same time, PEGylation markedly extends plasma half-life and systemic exposure, often by over 100- to 1000-fold [30,31]. Taken together, these findings indicate that an optimal PEG size must balance potency with pharmacokinetic stability. Among the derivatives evaluated, the 40 kDa PEG form provided this balance most effectively. In addition, 40 kDa PEG is the most widely approved high–molecular weight PEG in marketed pharmaceuticals, which further justifies its advancement as the lead candidate for development [31,32].

In the present study, plasma concentrations of PEG-AM on day 8 following administration of 25 nmol/kg PEG-AM were 94.7 ± 10.91 pmol/L (Figure 4e) and 96.3 ± 6.72 pmol/L (Figure 5f), which are approximately 10-fold higher than the effective blood concentrations of native AM observed in clinical trials for IBD [18]. Furthermore, when administered therapeutically after DSS-induced colitis onset, PEG-AM concentrations reached 473 ± 113 pmol/L at 25 nmol/kg and 12,911 ± 1363 pmol/L at 250 nmol/kg (Figure 6e), demonstrating that sufficient drug levels were achieved to exert a pharmacological effect.

PEGylation enhances protein stability [32,34]. Native AM is readily degraded by proteases, making it difficult to maintain effective plasma concentrations [35,36,37]. In contrast, PEG-AM remained highly stable in the serum, with 95.9% remaining after 60 min. We previously reported that native AM is degraded by thrombin in the serum [36], suggesting that PEGylation confers thrombin resistance. Although the identity of the plasma proteases responsible for native AM degradation remains unclear, PEGylation appears to confer broad protease resistance, contributing to sustained systemic exposure. Several studies have reported that AM binds to complement factor H [38,39]. Although such binding has been suggested to interfere with AM measurement, we confirmed that the addition of complement factor H did not affect the measurement of native AM (unpublished data). Nevertheless, further verification will be required for PEG-AM.

The extended half-life of PEG-AM is likely due to the bulky PEG moiety at the N-terminus, which increases molecular size, reduces renal clearance, and delays tissue distribution [40]. This mechanism is consistent with our previous work on AM derivatives conjugated to IgG1 Fc or human serum albumin (HSA), which also exhibited increased systemic retention [41,42]. An additional advantage often attributed to PEG-AM is its relatively lower immunogenicity compared with Fc- or HSA-conjugated derivatives, as PEGylation can sterically shield antigenic epitopes and thereby reduce immune recognition [43,44]. Furthermore, PEG-AM can be chemically synthesized, presenting challenges for large-scale production but allowing relatively straightforward clinical application. In contrast, Fc-fusion and HSA-conjugated variants require genetic engineering techniques; while scalable production is feasible, the initial manufacturing costs are prohibitively high for pharmaceutical development [45,46]. Therefore, a strategy of initially developing PEG-AM and subsequently transitioning to Fc-AM may represent a reasonable approach.

In the dose-dependent intravenous study (Figure 4), no statistically significant differences were detected in inflammation score, colon length, or colon weight-to-length ratio among groups. Nevertheless, the 25 nmol/kg dose was selected for further evaluation (Figure 5). This decision was based on several factors: (i) although not statistically significant, the 25 nmol/kg group showed the most favorable numerical values across multiple endpoints; (ii) previous studies demonstrated that both 20 kDa and 60 kDa PEG-AM exerted significant therapeutic effects at 25 nmol/kg when administered subcutaneously, supporting this dose as pharmacologically meaningful; and (iii) in our study, intravenous administration of 25 nmol/kg PEG-AM yielded plasma concentrations approximately ten-fold higher than the effective levels of native AM observed in prior clinical trials, suggesting clinical relevance. Thus, we proceeded with 25 nmol/kg PEG-AM in a larger cohort, where significant therapeutic effects were confirmed (Figure 5).

A single intravenous dose of 25 nmol/kg PEG-AM significantly improved the inflammation scores, bloody stool scores, and colon weight per unit length compared to the native AM. These improvements likely reflect the diverse biological actions of AM, including anti-inflammatory effects, promotion of epithelial repair, and modulation of immune responses [18,20,21], which are expected to be preserved in PEG-AM. Drug concentrations at the study endpoint were maintained at levels approximately 10 times higher than the peak concentration observed in prior AM trials for IBD [18], supporting its suitability for clinical application.

Although the detailed mechanism of action was not fully explored in this study, PEG-AM is presumed to act via the same receptor-mediated pathway as native AM. AM has been shown to ameliorate IBD through its anti-inflammatory activity and the promotion of epithelial repair, angiogenesis, and lymphangiogenesis [20,21,22]. Its direct regenerative effect on the intestinal epithelium, which is absent in current IBD therapeutics, has been highlighted as a major advantage in its therapeutic potential [22,23,24]. The combination of these diverse actions, including mucosal repair of the intestinal epithelium by AM, is considered to increase the likelihood of complete remission in patients with UC [18,20]. Considering that PEG-AM activates the same receptors, it is expected to exhibit similar clinical efficacy.

Receptor downregulation is a potential concern associated with high systemic drug concentrations [47,48]. Therefore, we examined the mRNA expression of AM and CGRP receptor components using quantitative PCR. Despite high plasma PEG-AM levels, no downregulation of the receptor components (RAMP1, RAMP2, RAMP3, or CLR) was observed. RAMP2 expression was significantly upregulated by 1.3-fold in the PEG-AM-treated group compared to the control (Figure 5h). A similar result was observed in the therapeutic efficacy study (Figure 6g); however, the difference between the treatment and control was not statistically significant. These findings should be interpreted with consideration of both statistical significance (*p* values and 95% CIs) and biological relevance (effect sizes) [49,50]. Importantly, receptor expression was not downregulated even at high systemic exposure levels, representing a novel finding of this study.

In DSS-induced colitis models, drugs are usually administered before or concurrent with DSS to evaluate prophylactic effects [21,30]. We tested the therapeutic potential of PEG-AM by initiating treatment five days after DSS exposure. Despite delayed administration, PEG-AM exhibited dose-dependent therapeutic effects. A higher dose (250 nmol/kg) was required for therapeutic efficacy than for prophylactic treatment (25 nmol/kg). Translating these doses to human equivalent doses (HED) using a standard conversion factor of 12.3 [50] suggests that the effective prophylactic and therapeutic doses in humans are approximately 2 nmol/kg and 20 nmol/kg, respectively. In a previous AM clinical trial, the dose required for remission induction was 16.8 nmol/kg [18], which is broadly comparable to the 20 nmol/kg estimated for remission induction with PEG-AM in this study. Furthermore, administration of 250 nmol/kg PEG-AM maintained plasma concentrations over 1000-fold higher than those reported in AM clinical studies, supporting the pharmacological activity of PEG-AM. These findings suggest that PEG-AM may be suitable for both the induction and maintenance of remission, although careful consideration of potential cardiovascular adverse effects such as hypotension will be essential in future clinical studies.

Despite these promising results, several limitations of this study are acknowledged. The in vivo pharmacological studies were conducted by a contract research organization (CRO), LSI Medience Ltd. (Kumamoto, Japan), and were not in an academic setting. Although the studies were conducted professionally, a blinded evaluation was not implemented, which may have introduced bias. Additionally, histological analysis of the colonic tissue was not performed. In our previous study on 20k PEG-AM, histological improvements were observed, but were not statistically significant [29]. Future pharmacological studies on PEG-AM should include blinded histopathological assessments to corroborate the current findings. Another limitation is that only male mice were used. There are reports suggesting that the effects of AM may differ between the sexes [51,52], so it will be important to verify the drug’s efficacy in female mice in future studies. Furthermore, a limitation of our study is that different molecular weights of DSS were used in the preventive and therapeutic models. Variations in DSS molecular weight are known to influence the severity and distribution of colitis [53,54]. In our design, this difference was intentional to establish a milder preventive model (MW 5000) and a more sustained therapeutic model (MW 36,000–50,000). Nevertheless, this factor should be considered when interpreting the comparative outcomes across studies.

The pharmacological effects of 40 k PEG-AM observed in our study are consistent with those of other studies conducted with 5 k, 20 k, and 60 k PEG-AM derivatives [29,30], indicating robustness of the data. Overall, our study findings support clinical development of PEG-AM as a novel therapeutic agent for IBD.

## 4. Materials and Methods

### 4.1. Peptide and Chemicals

Human AM, synthesized using the solid-phase method, was purchased from Peptide Institute Inc. (Osaka, Japan). A 40 kDa PEGylating reagent (SUNBRIGHT^®^ LY-400AL3) was obtained from NOF Corporation (Tokyo, Japan). PEG-AM was prepared as described by Roberts et al. [55]. The PEGylation site of PEG-AM was confirmed by ion-exchange chromatography with a CM-2SW 0.46 × 25 cm column (Tosho, Tokyo, Japan) following CNBr digestion as described previously [30]. PEG-AM was characterized by gel filtration chromatography using a Superdex 200 Increase 10/300 GL column (GE Healthcare, Little Chalfont, UK) and eluted with 20 mM trisodium citrate buffer (pH 7.2) containing 1.0 M NaCl. Absorbance was monitored at 280 nm. Human plasma (pooled with citric acid) and serum (pooled) were obtained from Cosmo Bio Co., Ltd. (Tokyo, Japan). Dextran sodium sulfate DSS (MW 5000) was purchased from Wako Pure Chemical Industries (Osaka, Japan) and DSS (MW 36,000–50,000) was obtained from MP Bio Japan K.K. (Tokyo, Japan).

### 4.2. In Vitro Biological Activity of PEG-AM

Human embryonic kidney (HEK)-293 cells stably expressed the AM type I receptor (AM1 receptor), as previously described [33]. This receptor subtype is highly specific to AM and consists of co-expressed calcitonin receptor-like receptor (CLR) and receptor activity-modifying protein 2 (RAMP2) [10,33]. Cells were maintained in Dulbecco’s Modified Eagle’s Medium (DMEM) supplemented with 10% fetal bovine serum, 100 U/mL penicillin, 100 μg/mL streptomycin, 0.25 μg/mL amphotericin B, 100 μg/mL hygromycin B, and 250 μg/mL geneticin at 37 °C in a humidified atmosphere of 95% air and 5% CO_2_. After three days of culture, 70–80% confluent cells were used for the intracellular cAMP stimulation assays. Cells were incubated with PEG-AM or AM in Hanks’ Balanced Salt Solution (Thermo Fisher Scientific K.K., Tokyo, Japan, #14060040) containing 0.2% bovine serum albumin (Sigma-Aldrich Japan, Tokyo, Japan, A4378-100G), 0.035% NaHCO_3_, and 0.5 mM isobutylmethylxanthine (Sigma-Aldrich Japan, I7018) for 15 min at 37 °C. The reactions were terminated with cell lysis buffer, and cAMP levels in the supernatants were measured using an enzyme immunoassay kit (GE Healthcare UK Limited, Little Chalfont, UK) [28].

### 4.3. Degradation of AM and PEG-AM

The reaction mixture consisted of 10 mM NaH_2_PO_4_ buffer (pH 7.1), 100 mM NaCl, and 100 μL of serum or plasma in a final volume of 200 μL. The degradation reaction was initiated by adding AM or PEG-AM and incubated at 37 °C. At specified time points, 30 μL aliquots were withdrawn, diluted in 170 μL of stop buffer, and stored at −80 °C until analysis.

### 4.4. Quantitative Analysis of AM and PEG-AM

AM and PEG-AM levels were measured using a fluorescence immunoassay (Tosoh Corporation, Tokyo, Japan) with two monoclonal antibodies: one recognizing the ring structure and the other targeting the amidated C-terminus of AM [27]. The assay showed 33% cross-reactivity with PEG-AM, 100% with native human AM, and 0% with native mouse AM on a molar basis.

### 4.5. Gene Expression of Receptor Components

After measuring colon length and weight, distal colon tissue was collected, immersed in RNAlater (Thermo Fisher Scientific K.K.), and stored at −80 °C until RNA extraction. Total RNA was extracted using the RNeasy Micro Kit (Qiagen, Singapore) and quantified using a DeNovix DS-11 Microvolume Spectrophotometer. cDNA was synthesized from 2 μg of total RNA using a high-capacity cDNA Reverse Transcription Kit (Thermo Fisher Scientific K.K.). Quantitative PCR was performed using the StepOnePlus Real-Time PCR System (Applied Biosystems, Waltham, MA, USA) and TaqMan Gene Expression Assays for mouse RAMP1, RAMP2, RAMP3, CLR (CALCRL), and GAPDH (internal control). Each 10 μL reaction included 5 μL of TaqMan Universal PCR Master Mix, 0.5 μL of gene-specific assay, 1 μL of 1:10 diluted cDNA, and nuclease-free water.

Thermal cycling was performed at 95 °C for 10 min, followed by 40 cycles of 95 °C for 15 s and 60 °C for 1 min.

All reactions were performed in duplicate. Relative expression levels were calculated using the 2^−ΔΔCt^ method [50] with GAPDH normalization.

### 4.6. Induction of DSS-Induced Colitis and PEG-AM Treatment

To evaluate the pharmacological effects of PEG-AM, three animal studies were conducted by the LSI Medience Corporation following the Animal Welfare Act and institutional approval. Seven-week-old male C57BL/6J JCL mice (CLEA Japan, Inc., Tokyo, Japan) were used in all studies and were housed under pathogen-free conditions.

#### 4.6.1. Dose-Dependent Study (Study No. P170454)

Colitis was induced with 3% (*w*/*v*) DSS (MW 5000) in drinking water for seven days. PEG-AM (1.0, 5.0, and 25 nmol/kg) or saline (control) was administered intravenously one day before DSS treatment. Each group included 10 mice. Stool consistency, bleeding, and body weight were evaluated on days −1, 0, 3, 5, and 7. A total inflammation score was calculated [22,36]. Mice were anesthetized with 3% isoflurane (Zoetis Japan, Tokyo, Japan), and the colon was excised on day 7.

#### 4.6.2. Efficacy of 25 nmol/kg PEG-AM (Study No. P180567)

Following the protocol described in Section 4.6.1, this study was conducted to confirm the efficacy of 25 nmol/kg intravenous PEG-AM. Each treatment group consisted of 20 mice.

#### 4.6.3. Therapeutic Effect After Disease Onset (Study No. P200487)

Colitis was induced with 2% (*w*/*v*) DSS (MW 36,000–50,000) for five days. On day 5, the mice were assigned to three groups and intravenously administered one of PEG-AM (25 or 250 nmol/kg) or saline. On day 6, DSS was replaced with water. Clinical symptoms were evaluated on days 5, 8, 10, and 12. The inflammation scores were calculated [30,56]. Colons were collected on day 12 under isoflurane anesthesia.

We used DSS with MW 5000 for preventive studies and MW 36,000–50,000 for therapeutic studies, as higher MW DSS is known to induce more sustained colitis suitable for therapeutic intervention experiments [53,54].

### 4.7. Statistical Analysis

Parametric data are presented as the mean ± SEM. Nonparametric data are presented as medians and were illustrated using violin plots. Statistical analyses were performed using GraphPad Prism version 8 (GraphPad Software Inc., La Jolla, CA, USA): One-way ANOVA followed by Steel’s (Figure 4b) or Dunnett’s tests (Figure 4c,d and Figure 6c,d). Two-way ANOVA followed by the Holm–Sidak post hoc test (Figure 6b). Mann–Whitney U test (Figure 5b,c). Unpaired *t*-test (Figure 5d,e,g–j). *p* < 0.05 was considered statistically significant.

## 5. Conclusions

PEG-AM retains potent in vitro biological activity and exhibits significant in vivo efficacy in a DSS-induced colitis model with a single intravenous dose providing both therapeutic and preventive effects. These findings suggest that PEG-AM is a promising therapeutic candidate for the treatment of patients with IBD.

## Data Availability

The datasets used and/or analyzed in the study are in the final report of each study. Data will be provided upon reasonable request and after execution of a confidentiality agreement. Further detailed information regarding the chemical properties, manufacturing, and control (CMC) of PEG-AM constitutes proprietary information and will be provided following the execution of a confidentiality agreement with Himuka AM Pharma Co., Ltd.

## Abbreviations

The following abbreviations are used in this manuscript:

AM	Adrenomedullin
PEG-AM	40 kDa PEGylated AM
cAMP	Cyclic adenosine monophosphate
DSS	Dextran sodium sulfate
UC	Ulcerative colitis
CD	Crohn’s disease
IBD	Inflammatory bowel disease
CGRP	Calcitonin gene-related peptide
CLR	Calcitonin receptor-like receptor
RAMP1	Receptor activity-modifying protein 1
RAMP2	Receptor activity-modifying protein 2
RAMP3	Receptor activity-modifying protein 3
DiPEG-AM	DiPEGylated adrenomedullin
5KPEG-AM	5 kDa PEGylated AM
20KPEG-AM	20 kDa PEGylated AM
60KPEG-AM	60 kDa PEGylated AM
HSA	Human serum albumin

## References

[1] Bretto E., Urpì-Ferreruela M., Casanova G.R., González-Suárez B. (2025). The Role of Gut Microbiota in Gastrointestinal Immune Homeostasis and Inflammation: Implications for Inflammatory Bowel Disease. Biomedicines.

[2] Bai J., Bouwknegt D.G., Weersma R.K., Dijkstra G., van der Sloot K.W.J., Festen E.A.M. (2025). Gene-Environment Interactions in Inflammatory Bowel Disease: A Systematic Review of Human Epidemiologic Studies. J. Crohn’s Colitis.

[3] Solitano V., Bernstein C.N., Dotan I., Dignass A., Domilici R., Dubinsky M.C., Gearry R.B., Hart A., Kaplan G.G., Ma C. (2025). Shaping the future of inflammatory bowel disease: A global research agenda for better management and public health response. Nat. Rev. Gastroenterol. Hepatol..

[4] Young J.C., Helsingen L.M., Refsum E., Högdén A., Perrin V., Løberg M., Gantzel R.H., Bretthauer M., Berglund A., Holme Ø. (2025). Temporal trends in characteristics and management of inflammatory bowel disease. Scand. J. Gastroenterol..

[5] Maksic M., Corovic I., Maksic T., Zivic J., Zivic M., Zdravkovic N., Begovic A., Medovic M., Kralj D., Todorovic Z. (2025). Molecular Insight into the Role of HLA Genotypes in Immunogenicity and Secondary Refractoriness to Anti-TNF Therapy in IBD Patients. Int. J. Mol. Sci..

[6] Sharma K., da Silva B.C., Hanauer S.B. (2025). The role of immunogenicity in optimizing biological therapies for inflammatory bowel disease. Expert Rev. Gastroenterol. Hepatol..

[7] Kitamura K., Kangawa K., Kawamoto M., Ichiki Y., Nakamura S., Matsuo H., Eto T. (1993). Adrenomedullin: A novel hypotensive peptide isolated from human pheochromocytoma. Biochem. Biophys. Res. Commun..

[8] Champion H.C., Nussdorfer G.G., Kadowitz P.J. (1999). Structure-activity relationships of adrenomedullin in the circulation and adrenal gland. Regul. Pept..

[9] Watanabe T.X., Itahara Y., Inui T., Yoshizawa-Kumagaye K., Nakajima K., Sakakibara S. (1996). Vasopressor activities of N-terminal fragments of adrenomedullin in anesthetized rat. Biochem. Biophys. Res. Commun..

[10] Fischer J.P., Els-Heindl S., Beck-Sickinger A.G. (2020). Adrenomedullin–Current perspective on a peptide hormone with significant therapeutic potential. Peptides.

[11] McLatchie L.M., Fraser N.J., Main M.J., Wise A., Brown J., Thompson N., Solari R., Lee M.G., Foord S.M. (1998). RAMPs regulate the transport and ligand specificity of the calcitonin-receptor-like receptor. Nature.

[12] Kamitani S., Asakawa M., Shimekake Y., Kuwasako K., Nakahara K., Sakata T. (1999). The RAMP2/CRLR complex is a functional adrenomedullin receptor in human endothelial and vascular smooth muscle cells. FEBS Lett..

[13] Pioszak A.A., Hay D.L. (2020). RAMPs as allosteric modulators of the calcitonin and calcitonin-like class B G protein-coupled receptors. Adv. Pharmacol..

[14] Alonso Martinez L.M., Harel F., Létourneau M., Finnerty V., Fournier A., Dupuis J., DaSilva J.N. (2019). SPECT and PET imaging of adrenomedullin receptors: A promising strategy for studying pulmonary vascular diseases. Am. J. Nucl. Med. Mol. Imaging.

[15] Dupuis J., Caron A., Ruel N. (2005). Biodistribution, plasma kinetics and quantification of single-pass pulmonary clearance of adrenomedullin. Clin. Sci..

[16] Bálint L., Nelson-Maney N.P., Tian Y., Serafin S.D., Caron K.M. (2023). Clinical Potential of Adrenomedullin Signaling in the Cardiovascular System. Circ. Res..

[17] Kita T., Kitamura K. (2022). Translational studies of adrenomedullin and related peptides regarding cardiovascular diseases. Hypertens. Res..

[18] Kita T., Ashizuka S., Ohmiya N., Yamamoto T., Kanai T., Motoya S., Hirai F., Nakase H., Moriyama T., Nakamura M. (2021). Adrenomedullin for steroid-resistant ulcerative colitis: A randomized, double-blind, placebo-controlled phase-2a clinical trial. J. Gastroenterol..

[19] Kita T., Ashizuka S., Takeda T., Matsumoto T., Ohmiya N., Nakase H., Motoya S., Ohi H., Mitsuyama K., Hisamatsu T. (2022). Adrenomedullin for biologic-resistant Crohn’s disease: A randomized, double-blind, placebo-controlled phase 2a clinical trial. J. Gastroenterol. Hepatol..

[20] Ashizuka S., Kita T., Inatsu H., Kitamura K. (2021). Adrenomedullin: A Novel Therapeutic for the Treatment of Inflammatory Bowel Disease. Biomedicines.

[21] MacManus C.F., Campbell E.L., Keely S., Burgess A., Kominsky D.J., Colgan S.P. (2011). Anti-inflammatory actions of adrenomedullin through fine tuning of HIF stabilization. FASEB J..

[22] Hayashi Y., Narumi K., Tsuji S., Tsubokawa T., Nakaya M.A., Wakayama T., Zuka M., Ohshima T., Yamagishi M., Okada T. (2011). Impact of adrenomedullin on dextran sulfate sodium-induced inflammatory colitis in mice: Insights from in vitro and in vivo experimental studies. Int. J. Color. Dis..

[23] Kawaguchi M., Kataoka H., Kiwaki T., Weiting L., Nagata S., Kitamura K., Fukushima T. (2023). Adrenomedullin alleviates mucosal injury in experimental colitis and increases claudin-4 expression in the colonic epithelium. FEBS Open Bio.

[24] Yi Z., Fan H., Liu X., Tang Q., Zuo D., Yang J. (2015). Adrenomedullin improves intestinal epithelial barrier function by downregulating myosin light chain phosphorylation in ulcerative colitis rats. Mol. Med. Rep..

[25] Geven C., Kox M., Pickkers P. (2018). Adrenomedullin and Adrenomedullin-Targeted Therapy As Treatment Strategies Relevant for Sepsis. Front. Immunol..

[26] Kataoka Y., Miyazaki S., Yasuda S., Nagaya N., Noguchi T., Yamada N., Morii I., Kawamura A., Doi K., Miyatake K. (2010). The first clinical pilot study of intravenous adrenomedullin administration in patients with acute myocardial infarction. J. Cardiovasc. Pharmacol..

[27] Kita T., Kaji Y., Kitamura K. (2020). Safety, Tolerability, and Pharmacokinetics of Adrenomedullin in Healthy Males: A Randomized, Double-Blind, Phase 1 Clinical Trial. Drug Des. Dev. Ther..

[28] Akashi E., Nagata S., Yamasaki M., Kitamura K. (2020). Activation of Calcitonin Gene-Related Peptide and Adrenomedullin Receptors by PEGylated Adrenomedullin. Biol. Pharm. Bull..

[29] Miki G., Kuroishi N., Tokashiki M., Nagata S., Tamura M., Yoshiya T., Yoshizawa-Kumagaye K., Ashizuka S., Kato J., Yamasaki M. (2020). 20 kDa PEGylated Adrenomedullin as a New Therapeutic Candidate for Inflammatory Bowel Disease. Gastrointest. Disord..

[30] Nagata S., Yamasaki M., Kitamura K. (2017). Anti-Inflammatory Effects of PEGylated Human Adrenomedullin in a Mouse DSS-Induced Colitis Model. Drug Dev. Res..

[31] Gao Y., Joshi M., Zhao Z., Mitragotri S. (2024). PEGylated therapeutics in the clinic. Bioeng. Transl. Med..

[32] Zhang J., Guo S., Wang T., Chen Q. (2025). Comparison between long-acting pegylated and daily recombinant human growth hormone for pediatric growth hormone deficiency a systematic review. Sci. Rep..

[33] Kuwasako K., Shimekake Y., Masuda M., Nakahara K., Yoshida T., Kitaura M., Kitamura K., Eto T., Sakata T. (2000). Visualization of the calcitonin receptor-like receptor and its receptor activity-modifying proteins during internalization and recycling. J. Biol. Chem..

[34] Hung K.Y., Kowalczyk R., Desai A., Brimble M.A., Marshall J.F., Harris P.W.R. (2022). Synthesis and Systematic Study on the Effect of Different PEG Units on Stability of PEGylated, Integrin-αvβ6-Specific A20FMDV2 Analogues in Rat Serum and Human Plasma. Molecules.

[35] Corti R., Burnett J.C., Rouleau J.L., Ruschitzka F., Lüscher T.F. (2001). Vasopeptidase inhibitors: A new therapeutic concept in cardiovascular disease?. Circulation.

[36] Nishimoto Y., Nagata S., Akashi E., Yamasaki M., Kitamura K. (2020). Thrombin rapidly digests adrenomedullin: Synthesis of adrenomedullin analogs resistant to thrombin. Biochem. Biophys. Res. Commun..

[37] Schonauer R., Els-Heindl S., Fischer J.P., Kobberling J., Riedl B., Beck-Sickinger A.G. (2016). Adrenomedullin 2.0: Adjusting Key Levers for Metabolic Stability. J. Med. Chem..

[38] Pío R., Elsasser T.H., Martínez A., Cuttitta F. (2002). Identification, characterization, and physiological actions of factor H as an adrenomedullin binding protein present in human plasma. Microsc. Res. Tech..

[39] Pio R., Martinez A., Unsworth E.J., Kowalak J.A., Bengoechea J.A., Zipfel P.F., Elsasser T.H., Cuttitta F. (2001). Complement factor H is a serum-binding protein for adrenomedullin, and the resulting complex modulates the bioactivities of both partners. J. Biol. Chem..

[40] Harris J.M., Chess R.B. (2003). Effect of pegylation on pharmaceuticals. Nat. Rev. Drug Discov..

[41] Kuroishi N., Nagata S., Akashi E., Ashizuka S., Kato J., Yamasaki M., Kitamura K. (2021). Development of a novel human adrenomedullin derivative: Human serum albumin-conjugated adrenomedullin. J. Biochem..

[42] Nagata S., Yamasaki M., Kuroishi N., Kitamura K. (2022). Development of Long-Acting Human Adrenomedullin Fc-Fusion Proteins. Biology.

[43] Chen B.M., Cheng T.L., Roffler S.R. (2021). Polyethylene Glycol Immunogenicity: Theoretical, Clinical, and Practical Aspects of Anti-Polyethylene Glycol Antibodies. ACS Nano.

[44] Li C., Li T., Tian X., An W., Wang Z., Han B., Tao H., Wang J., Wang X. (2024). Research progress on the PEGylation of therapeutic proteins and peptides (TPPs). Front. Pharmacol..

[45] Chuang V.T., Kragh-Hansen U., Otagiri M. (2002). Pharmaceutical strategies utilizing recombinant human serum albumin. Pharm. Res..

[46] Zeng L., Yang K., Wu Y., Yu G., Yan Y., Hao M., Song T., Li Y., Chen J., Sun L. (2024). Telitacicept: A novel horizon in targeting autoimmunity and rheumatic diseases. J. Autoimmun..

[47] Hosoda H., Nakamura T., Yoshihara F. (2022). Plasma Clearance of Intravenously Infused Adrenomedullin in Rats with Acute Renal Failure. Biomolecules.

[48] Kasahara T., Tanaka M., Zhao Y., Kamiyoshi A., Sakurai T., Ichikawa-Shindo Y., Kawate H., Matsuda Y., Zhang Y., Guo Q. (2024). Receptor activity-modifying proteins of adrenomedullin (RAMP2/3): Roles in the pathogenesis of ARDS. Peptides.

[49] Bustin S.A., Benes V., Garson J.A., Hellemans J., Huggett J., Kubista M., Mueller R., Nolan T., Pfaffl M.W., Shipley G.L. (2009). The MIQE guidelines: Minimum information for publication of quantitative real-time PCR experiments. Clin. Chem..

[50] Livak K.J., Schmittgen T.D. (2001). Analysis of relative gene expression data using real-time quantitative PCR and the 2(-Delta Delta C(T)) Method. Methods.

[51] Armstead W.M., Kiessling J.W., Bdeir K., Kofke W.A., Vavilala M.S. (2010). Adrenomedullin prevents sex-dependent impairment of autoregulation during hypotension after piglet brain injury through inhibition of ERK MAPK upregulation. J. Neurotrauma.

[52] Trincot C.E., Xu W., Zhang H., Kulikauskas M.R., Caranasos T.G., Jensen B.C., Sabine A., Petrova T.V., Caron K.M. (2019). Adrenomedullin Induces Cardiac Lymphangiogenesis After Myocardial Infarction and Regulates Cardiac Edema Via Connexin 43. Circ. Res..

[53] Halasi M., Grinstein M., Adini A., Adini I. (2022). Fibromodulin Ablation Exacerbates the Severity of Acute Colitis. J. Inflamm. Res..

[54] Koboziev I., Karlsson F., Zhang S., Grisham M.B. (2011). Pharmacological intervention studies using mouse models of the inflammatory bowel diseases: Translating preclinical data into new drug therapies. Inflamm. Bowel Dis..

[55] Roberts M.J., Bentley M.D., Harris J.M. (2002). Chemistry for peptide and protein PEGylation. Adv. Drug Deliv. Rev..

[56] Hamamoto N., Maemura K., Hirata I., Murano M., Sasaki S., Katsu K. (1999). Inhibition of dextran sulphate sodium (DSS)-induced colitis in mice by intracolonically administered antibodies against adhesion molecules (endothelial leucocyte adhesion molecule-1 (ELAM-1) or intercellular adhesion molecule-1 (ICAM-1)). Clin. Exp. Immunol..

